# A MINE Alternative to D-Optimal Designs for the Linear Model

**DOI:** 10.1371/journal.pone.0110234

**Published:** 2014-10-30

**Authors:** Amanda M. Bouffier, Jonathan Arnold, H. Bernd Schüttler

**Affiliations:** 1 Institute of Bioinformatics, University of Georgia, Athens, Georgia, United States of America; 2 Genetics Department, University of Georgia, Athens, Georgia, United States of America; 3 Physics and Astronomy Department, University of Georgia, Athens, Georgia, United States of America; University of East Piedmont, Italy

## Abstract

Doing large-scale genomics experiments can be expensive, and so experimenters want to get the most information out of each experiment. To this end the Maximally Informative Next Experiment (MINE) criterion for experimental design was developed. Here we explore this idea in a simplified context, the linear model. Four variations of the MINE method for the linear model were created: MINE-like, MINE, MINE with random orthonormal basis, and MINE with random rotation. Each method varies in how it maximizes the MINE criterion. Theorem 1 establishes sufficient conditions for the maximization of the MINE criterion under the linear model. Theorem 2 establishes when the MINE criterion is equivalent to the classic design criterion of D-optimality. By simulation under the linear model, we establish that the MINE with random orthonormal basis and MINE with random rotation are faster to discover the true linear relation with 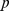 regression coefficients and 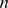 observations when 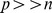. We also establish in simulations with 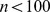, 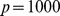, 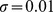 and 1000 replicates that these two variations of MINE also display a lower false positive rate than the MINE-like method and additionally, for a majority of the experiments, for the MINE method.

## Introduction

The Problem: The researcher wishes to carry out model-guided discovery about a system from a sequence of 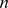 experiments. The challenge is that each of the 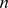 experiments performed is very expensive, and so at each stage 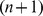 it is desirable to design the next experiment to be maximally informative. The approach in which 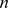 experiments are to be done sequentially in such a way as to capture the most information at each stage n about the underlying model is called utilizing the Maximally Informative Next Experiment (MINE) [1]. The method has been shown to be consistent for one version of MINE [2]. To understand MINE we will consider the linear model, 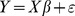, where 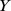 is a 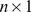 vector of dependent measurements, 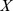 is a 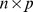 matrix of 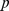 independent variables, each with 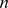 measurements, 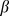 is a 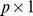 parameter vector, and 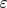 is a 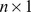 vector of independently and identically distributed normal 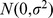 errors.

**Figure 1:**
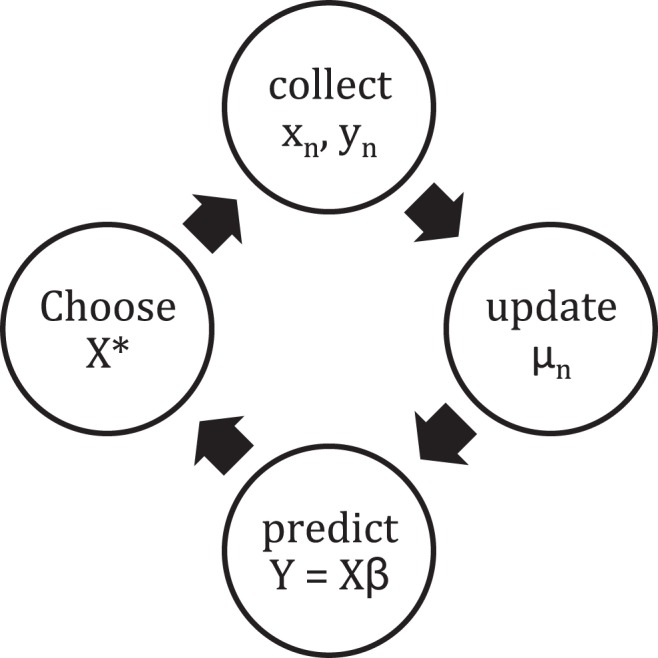
Cycle of MINE discovery – Simplified Computing Life Paradigm.

The problem has four features. First, there are many parameters and limited data 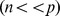 so there will be many more unknown parameters than data. In this setting a large sample of variables 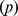 is to be observed as it is not known in advance which ones are relevant. In fact, typically 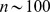 while 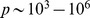. Second, the 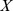 matrix is partitioned into two parts, 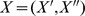, where 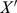 is an 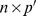 matrix of independent variables that the experimentalist can control and 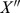 is an 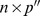 matrix of independent variables that cannot be controlled under the conditions 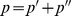 [3]. The 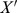 matrix will be referred to as the design matrix. For simplicity, we will assume the entire 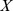 matrix is made up of 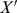. Third, the next experiment encompasses stages 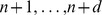, where 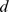 is the dimension of the experiment. Experiments constitute batches of 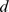 observations. The fourth and final feature of the experiment is that each experiment of 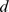 observations is very costly, be it time, materials and/or subjects, or financially such as $250,000 per experiment [4]. So at each stage 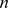 in the overall study, there is a high premium on choosing the best next experiment. The problem is to discover with reasonably high probability the model 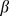 in as few steps 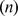 as possible. We call this a problem in model discovery because what we wish to know is what linear relation can be discovered from the many variables 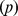 measured over the time course of the study. Again the number of variables measured is large because it is not known in advance which ones are relevant. The process of discovering the model is cyclical as shown in Figure 1.

This problem makes points of contact with several distinguished problems in statistics and engineering. There are problems in experimental design [5], [6], [7], [8], [9] leading to model refinement with n>p, particularly for sequential designs [10]. There are problems in control, as addressed by, for example, with response surfaces [11]. The problem of model guided discovery, we will show, is distinct from all of these because it is focused on discovery (in this case the unknown linear relation).

As an example problem for use in model-guided discovery, suppose the researcher wishes to understand human longevity [12]. The researcher may examine the characteristics of US centenarians. Several thousand variables are measured including genetics, diet, and lifestyle on each centenarian because it is unknown which variable or variables have an effect on longevity. Some of these variables can be controlled, such as diet and lifestyle. Others, like the genes carried by the centenarian, cannot be controlled. In model refinement, the goal is to select a design, 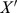 (diet and lifestyle), to reduce the error in the parameters 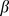 by consideration of, for example, 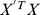 [7] and its determinant (*i.e*. D-optimality). In process control with the aid of response surfaces, the goal might be to select a design 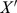 to maximize the expected longevity 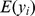 (where 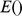 denotes expectation and 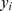 denotes the longevity of the i^th^ individual in the study) by manipulating diet and lifestyle. Such an engineering approach to extending lifespan has been implemented in the nematode [13]. In model-guided discovery the goal is simply to choose a design 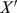 at each stage to discover the factors that determine longevity with as few centenarians 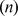 as possible and using limited data, to discover potentially many important variables.

Another example of this framework in systems biology can be seen in the description of genetic networks at a steady state or system in equilibrium [14]. In this setup a genetic network is approximated to first order by the following linear system:
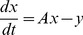
(1)


Here the column vector 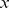 describes the concentration of mRNAs of genes in a network, and the 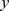 vector describes external perturbations. The 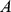 matrix captures the network relationships among the genes, and 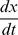 is the derivative with respect to time.

A steady state is assumed so that the dynamical system reduces to:
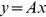
(2)


The problem is to infer the network 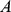. An experiment entails measuring all mRNAs under a particular perturbation y, so several perturbations are tested. This setup reduces to a linear regression problem. Such design problems have been considered for nonlinear genetic network models as well [4], [8], [15], [16], but we will not focus on these here.

## Mathematical Results

### Model Estimation by the Ensemble Method2

A standard approach to estimating the regression coefficients 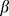 is the least squares method. This approach reduces to solving the normal equations below for the least squares estimates of the parameters 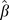:
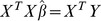
(3)


The challenge in our problem is that 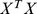 will not often be of full rank because of collinearity in the independent variables and because there are so few data points relative to the number of parameters 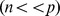. While the normal equations in (3) could be solved by use of a generalized inverse, there are likely to be many solutions that are equally consistent with the data and not one best least squares estimate 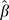 of the parameters in the model. The key is to not find one estimate, but rather an ensemble of estimates consistent with the data 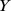.

To address this problem the likelihood is consulted at each stage:
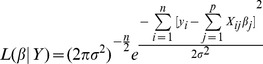
(4)


Since there are so many independent variables with so little data, this surface resembles a golf course with its varied terrain of many hills and sandpits than a mountain or mountain range. However, we can reconstruct the entire likelihood function by Markov Chain Monte Carlo methods (MCMC). By integrating over a standardized L(β) with respect to 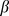 and using a particular prior distribution, we can make predictions about the behavior of the system even in the presence of such limited data [17]. So instead of finding one best parameter 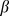 to represent the system, instead we construct 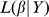 or alternatively, the entire posterior distribution with a different prior distribution. These are special cases of the ensemble method, in which some figure of merit is used to select a distribution of models fitting experimental data [17], and as a special case the reconstruction of the standardized 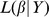 is referred to as the ensemble. In this paper the standardized likelihood can be calculated exactly when the variance 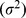 is known, the case to be used here, and with a Gaussian conjugate prior on 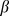 from Eqn (4) (see ref [18]):
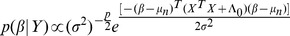
(5)


The posterior mean µ_n_ is described as:
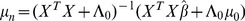
(6)


The least squares estimate 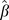 enters into the calculation of the posterior mean 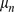. In the program description section below, we replace 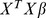 in (6) with 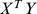 from (3).

Here the precision matrix which determines the prior distribution in (5) is 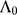 and will be taken as 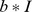 where 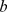 is a positive constant and 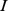 is the 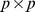 identity matrix. We also refer to this (5) as an example of the ensemble 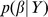. In the past we have used a uniform prior over a finite interval [17], but the Gaussian prior distribution insures that the integration can be done along all components of the parameter vector 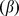 over the whole parameter space and can approach that of a noninformative prior distribution by letting diagonal elements of this matrix become small.

Normally the moments of the ensemble would be calculated by MCMC methods [17], but from (5) we can obtain the moments of 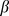 directly and for example the linear model with known error variance.
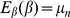
(6)

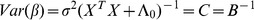
(7)


These moments of the ensemble can be updated as each observation is added.

### Maximally Informative Next Experiment

At each stage 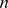, we choose the next experiment 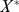 by reference to the ensemble 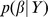 in (5) to infer the unknown but true regression parameters 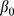. The new design matrix consists of 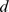 rows, and after completion of the next experiment is used to augment 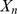 to 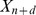 or 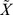. The design of the new experiment is captured in 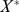 and the augmented/updated design for all experiments in 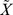. For each member of the ensemble 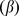 we make a prediction vector about the 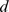 outcomes 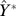 of the next experiment, namely 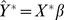, where 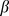 is drawn from the ensemble of models in (5), 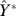 is the vector of 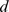 predictions for the next experiment, and 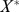 is the design of the next experiment. We choose the new experiment 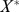 such that we have maximum discrimination between the alternatives in the ensemble. If two random models of the ensemble should have correlated predicted responses 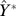 for experiment 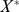, the choice of design would be poor as this would not reveal as much information as when two random members should have uncorrelated responses.

One MINE criterion for choice of 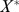 was developed by use of a microscope analogy [4]. The object in the microscope is 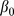. The image under the microscope 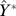 is mapped onto the object 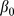 in the field of the microscope, but the mapping is fuzzy and imperfect with their being uncertainty in 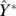. Let 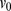 be a volume in the object space (*i.e*., the parameter space 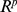) under the light microscope where 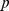 is the number of parameters, and let 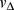 be the “image difference volume” swept out that is viewed. For a microscope, the connection between the two volumes is the model of physical optics. In our context here, the connection is the model prediction 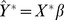. Formally, the image difference volume 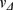 is swept out by the image difference vector 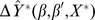 for all pairs of objects 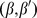 in 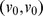 or
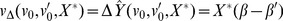
(8)


The image difference volume is swept out by varying 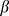 and 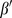 in (8).

The image difference volume depends explicitly on 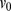 and how we “twiddle the dials” on the microscope though 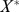. The maximally informative next experiment (MINE) criterion is based on this idea that the more volume in 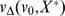, the more detail discerned in 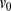. This is achieved by adjusting the data captured in 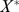.

In order to take advantage of this MINE criterion, we must make a selection of the object volume 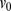. The choice is improvised but driven by computational practicality [4]; other choices are possible [19]. We elect to define a “representative volume” 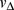 swept out by 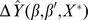 when 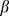 and 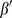 are drawn randomly as “typical” or average values from the ensemble pair distribution 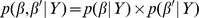, the components given by (5). The volume is constructed from the variance – covariance ellipsoid of the image difference volume 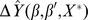 and is dependent on the choice of experiment 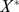. We define the ensemble distribution of 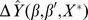 as:
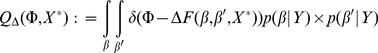
(9)


The quantity 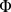 is any point in the 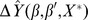 volume and 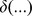 is the Dirac Delta Function. The ensemble distribution in (9) specifies an effective difference volume 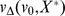 in the image difference space 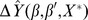 by way of the characteristic ellipsoid of this space specified by:
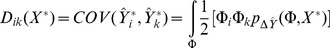
(10)


The variance – covariance ellipsoid is centered at the origin, 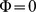 because 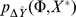 is an even function in 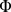 due to 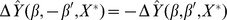. The variance – covariance ellipsoid has the D-matrix eigenvalues and directions of the half-axes given by the D-matrix eigenvectors. From (10) we can write the covariance ellipsoid in terms of the moments of the ensemble:
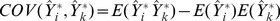
(11)


Normally these moments could be computed by MCMC methods [17], but because of the explicit form in (5) we can evaluate (11) directly from (5) as:
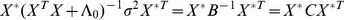
(12)


The matrix  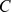 is defined to be 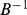. A Hilbert Space (HS, *i.e*., a complete inner product space) formalism is introduced to give a compact form to the MINE criterion. The HS of functions consist of functions defined on the model parameter space, 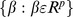, for which the covariance is the HS inner product. This inner product is formally defined as:
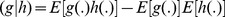
(13)


The components of the observation vector, 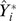, are represented by:
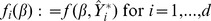
(14)


We can write the covariances in terms of the inner product:
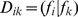
(15)


The ensemble standard deviation of the prediction 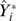 is equivalent to the HS vector norm or length denoted by 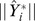. The norm is defined by 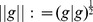


If the predictions 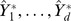 are linearly dependent, then the HS prism is defined by the predictions collapses to a lower dimensional one, and the determinant 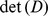 vanishes. If the predictions are not linearly dependent, then predictions determine an HS prism whose volume is simply given by the product of their vector lengths, namely 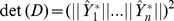. In general the predictions are correlated, and we have the Hadamard Inequality:
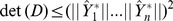
(16)


The ratio 
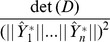
 can be thought of as a composite measure of the dependence of the predictions and is a function only of the HS angles between predictions.

We are now in a position to introduce a MINE criterion first by introducing the normalized predictions.
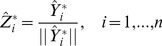
(17)


The normalized covariance matrix or correlation matrix denoted by R is defined by:
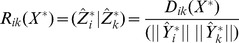
(18)

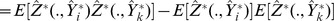



This is the correlation matrix among the predictions. We propose the following MINE design criterion 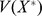:
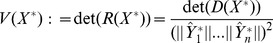
(19)


This criterion is the squared volume of a prism spanned by the normalized predictions 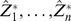. Such a criterion is advantageous when the predictions are almost but not actually/completely linearly dependent. This is a situation that has been encountered in practice [4]. This MINE criterion from (12) only depends on the ensemble through its variance-covariance matrix and not its mean in (6). The MINE criterion is also scale-free [4], [20]. It clearly differs from the usual model refinement criterion based on 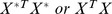.

In practice the MINE criterion will behave better than 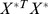 for 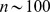 and 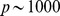 because its calculation through inverting 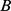 is stabilized by 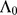 in (12), as in Ridge Regression [21] and will potentially incorporate the data from prior experiments in (12) through the 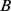 matrix. Its form also lends itself to optimization for large problems as will be shown under Theorem 1 below and under simulation results later 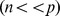.

### Maximizing the MINE Criterion

Ideally we would have a necessary and sufficient condition for maximizing the MINE criterion. Here in Theorem 1 we only present a sufficient condition for maximizing the MINE because the necessary condition has not been found.

**Theorem 1:** If the rows 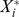 of the design matrix 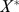 are chosen to be 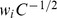, where 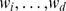 is any orthonormal set, then the MINE criterion 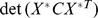 is maximized.

Proof: 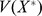 is maximized when 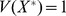. This occurs if and only if 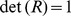 which is only satisfied if and only if 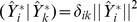 from (19), where 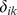 is the Kroneker delta. From (13) the inner product can be used to represent the covariances as 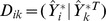. The condition 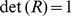 is thus equivalent to 
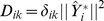
 or equivalently 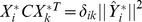 (The 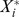 denotes the i^th^ column of 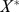). We now need to introduce two more equivalencies: 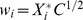 and 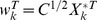. The fact that any positive semi-definite symmetric matrix, such as 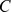, has a square root gives us the liberty to create such a 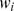. Since 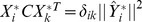, this leads to 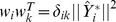, which implies any orthonormal basis can be used for 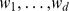.

In particular, the vectors 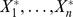 can be selected as the eigenvectors of 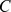 once standardized by 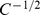. One efficient route for maximizing the MINE criterion is then simply to compute the eigenvectors and eigenvalues of 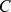 or equivalently, to maximize the parallelpiped whose volume is 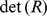 [4] and then to normalize them by 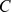 in 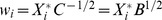. The choice of normalization still needs to be examined as a model-guided discovery tool. See simulation results for examination of three choices of different orthonormal bases, a (1) normalized eigenvector basis; (2) random basis; (3) normalized eigenvector basis with random rotation.

### Model Refinement

A traditional approach to choosing the design 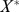 (in contrast to MINE) is to choose 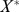 to maximize some simple function of the variance-covariance matrix of the parameter estimates 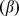 such as the determinant, to create a D-optimal design [6], [8]. Consider then the augmented design matrix 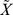 which is not only a function of the current design 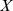 but includes the possible design of the new experiment 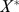. This means:
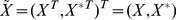
(20)


Under ordinary model refinement, we wish to minimize some simple function of the variance-covariance matrix of 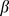, such as:
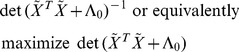
(21)


This can be written explicitly in terms of the new experiment with the identity:
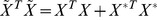
(22)


The model refinement criterion is then to maximize 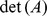 where:
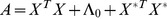
(23)


The derivative of 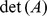 with respect to each component of 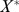 can be computed from:
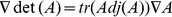
(24)


Here the 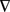 is the gradient with respect to 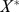, 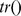 refers to the trace and 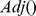 denotes the Adjoint. A necessary condition for maximizing the 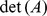 is for 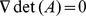. This max determinant (max det) problem is closely related to solutions to an affine formulation of this max det problem, and the problem is most closely related to the *analytic centering problem* [22]. These authors cast the search for D-optimality in design as a convex optimization problem with the max det problem linear in 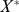 and with linear inequality constraints [22]. The linearity in 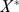 is achieved by constructing 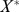 from a set of rows (or designs) that are known in advance. The optimization problem is then reduced to determining how often each row (design) is used. Here we do not know the rows in advance.

### MINE can produce a D-optimal Design

Kiefer and Wolfowitz [23] established that D-optimal designs are equivalent to mini-max designs, which minimize the maximum of the expected loss associated with each possible design. It is natural to ask whether or not there is any such relation between D-optimal designs and MINE. While the model refinement procedure appears to start from an entirely different criterion than MINE, it is possible to establish a relation between these different kinds of optimal designs by imposing the same constraints on the respective optimization problems. When we do this, we can establish:

**Theorem 2 (Equivalence Theorem of D-optimality and MINE):** The MINE procedure in Theorem 1 is D-optimal in the sense that 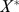 maximizes 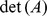 subject to the constraint 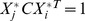 where 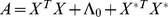.

Proof: In order for MINE and a D-optimal solution to be directly comparable they need to be maximized subject to the same constraints on 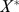. So we maximize 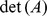 subject to the following constraint from (12):
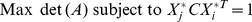
(25)


The constraint insures the columns of 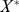 are an orthonormal basis.

We can introduce a related criterion 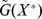 as:
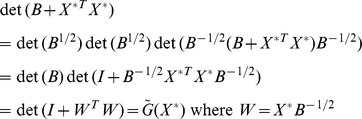



Note that 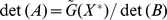. So maximizing 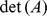 is the same as maximizing 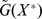. The maximization problem in (25) is equivalent to:
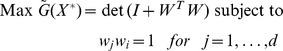
(26)


The constant 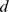 is the number of observations in the new experiment. We can think of the original optimization problem as equivalent to determining the best set of normalized vectors 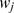.

From (26), the constraints imply that 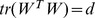 where 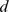 is the dimension of the next experiment (*i.e*., the number of observations in the next experiment). We also have the trace being the sum of the eigenvalues of 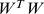.
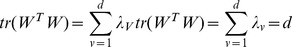
(27)


Constraint (27) implies a constraint on the eigenvalues in (27), but not the converse. To finish the proof we will first maximize 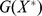 subject only to (27) reminiscent of [22].

We will then show the solution of this max det problem can also be chosen to satisfy all of the constraints in (26).
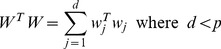
(28)


Since 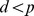, we can choose at least 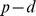 orthonormal vectors 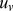 such that:
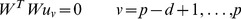
(29)


We choose these 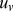 vectors also to be orthogonal to 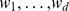. We will call this subspace of the parameter space as the unexplored subspace. Note that while 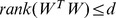, but dimension 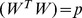 (that is, 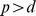 here). This implies that 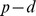 eigenvalues are zero. (This implies that for 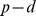 eigenvalues, say for 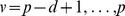, are zero. As an example, if 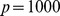 and 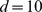, then the last 990 of the eigenvalues are zero. We have that:
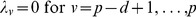
(30)


These degenerate eigenvalues in (30) are associated with the unexplored subspace. This fact along with the determinant being the product of the eigenvalues implies from (26):
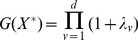
(31)


Now maximize 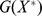 with respect to 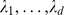 only subject to constraint [27] using the method of Lagrange multipliers (with multiplier 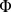). We find that:
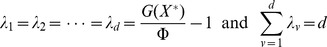
(32)


These two imply that 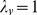 for 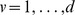 on the explored subspace of the parameter subspace.

The result of maximizing with respect to the eigenvalues is that 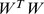 is diagonalized with only the first 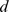 diagonal elements being 1. The maximum value of 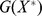 is 2^d^ from (31).

From here, if we choose 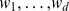 to be an orthonormal set such that 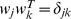, then we have 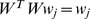 for all 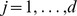. Thus 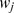 is an eigenvector of 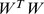 with eigenvalue 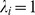 for 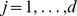. All constraints in (26) are satisfied for the solution to the max det problem with (27).

### Choice of prior distribution

The choice for the prior mean vector is reasonably taken as zero since most of the independent variables are not expected to have an effect on the dependent variable y. The only question is the choice of b specifying the precision matrix in 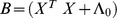 where 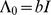 (and specifies the prior). Dumouchel and Jones [24] provide one argument to select b with an idea to making the design robust to violations of linear model assumptions. We will suggest another approach.

Let X^T^X have eigenvalues λ_i_ with corresponding orthonormal eigenvectors u_i_. Then we can write X^T^X and B as:
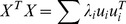
(33)




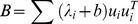
(34)

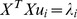
(35)

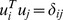
(36)


The eigenvalues of 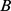 are 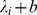 and have the same eigenvectors as 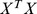. We can now introduce a new variable:
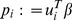
(37)


We can loosely think of the uncertainty or standard deviation of the 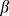-vectors (across the ensemble) in the 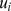 direction as:
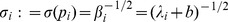
(38)


In the absence of any experimental data 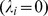, the uncertainty in the 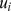 direction should reduce to:
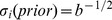
(39)


We would expect the uncertainty without experimental constraints (of data) to exceed the uncertainties with data or that:
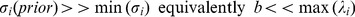
(40)


The maximum eigenvalue of 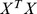 provides an upper bound on 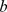. This one would be satisfied, for example, if 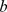 were chosen to be equivalent to the weight of one observation in 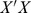. The matrix 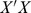 would quickly dominate. The next constraint is more stringent, and so it is not necessary to check that (40) is satisfied.

Another constraint on 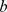 arises from the requirement that the true regression coefficients not be too far out in the tails of the prior distribution; otherwise the data through 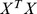 will never find the true regression coefficients. We can think of the prior distribution as equivalent to a fishing-net. We want this net to be well cast to catch the fish.

Introduce 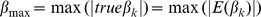 where the expectation is taken over the ensemble. Then the b-value should be chosen so that
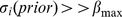
(41)


This is equivalent to requiring:
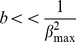
(42)


So with the prior data, 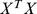, and some idea of the magnitude of the regression coefficients, there are constraints on the prior distribution as specified by the precision matrix and hence 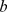. These constraints are satisfied in the simulations to follow. As an example, if the largest magnitude of a regression coefficient were 50, then 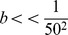 or 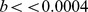. Since we set all variables and know the largest regression coefficient’s magnitude is 50, we set 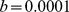. This is a tighter constraint than the first. We also do not want 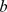 to be too small to allow 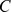 in (12) to still be inverted as in ridge regression.

## Methods

### Methods for simulating four versions of MINE under the linear model

**Figure 2:**
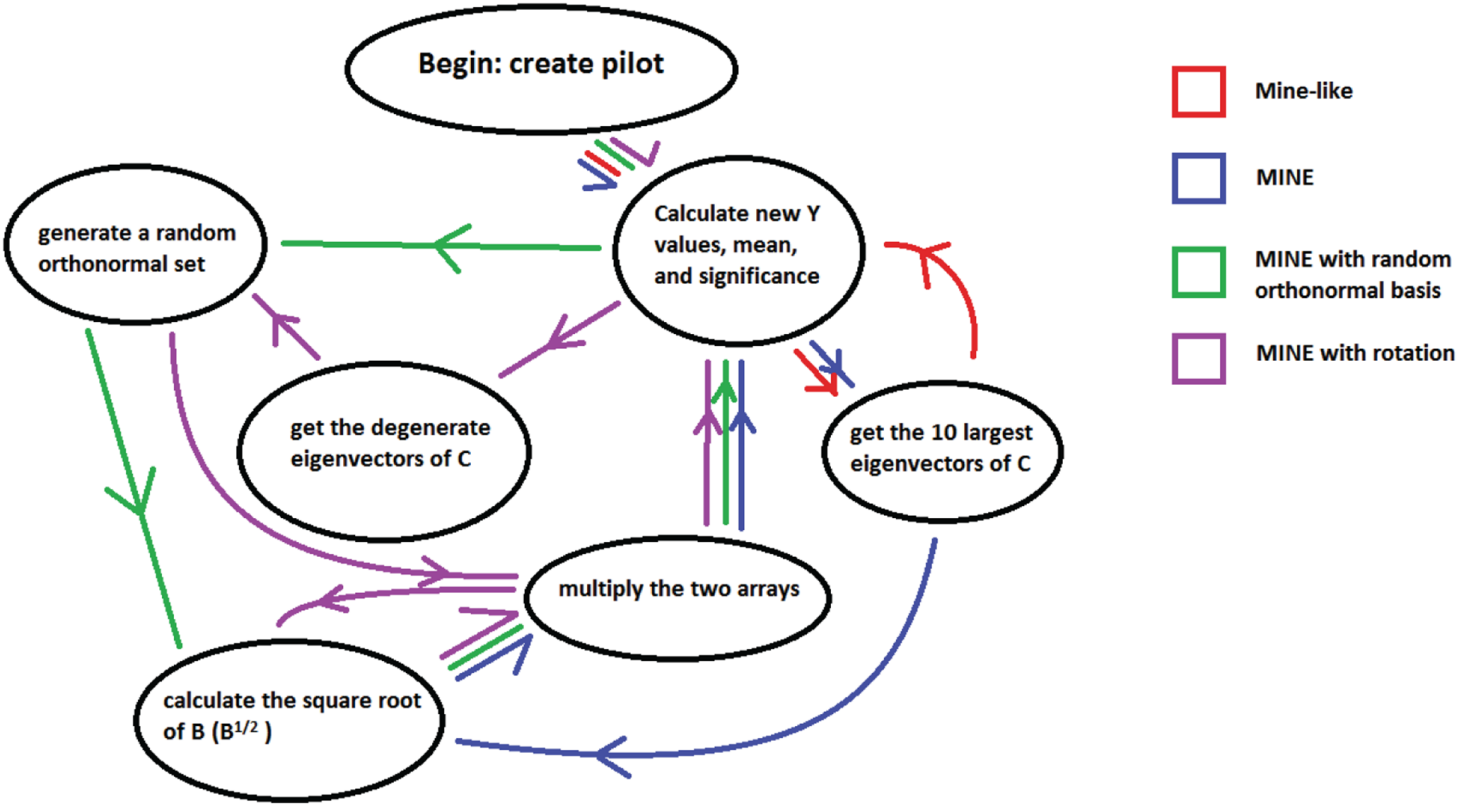
Visual representation of the pathways for each MINE method.

The program MINE to simulate the above procedures is written in Java under version 1.6 and utilizes the version 5 of the Jama library [25]. Details of the input and output of this software are already reported [26]. The program is available in sourceforge.net under the name linearminesimulations. There are four variants on the MINE method described below in this section and summarized in Figure 2.

Following the initialization of many variables (including but not limited to the matrices and vectors to store the 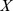 matrix, 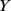 matrix and *ε*, the number of variables 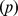 and the significance matrix) and the set up of data from the input file, the main part of the simulation program begins. Since the first experiment has no data on which to base a design, this pilot experiment is randomly generated. The simulation program is designed to handle a variety of data. If the number of variables is over 50, then the first experiment is selected to have 10 observations; otherwise, the first experiment has between five and nine observations. The number of pilot observations varies between 0 and 10 as determined by the number of variables. Each value in the pilot is created by generating a random number between 0 and 10 and then dividing it by the 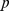 value. The set is not then normalized nor orthogonal. After the pilot experiment is generated, the random number generator is changed depending on which method is used in order to allow for the same pilots but the rest of the numbers generated being different. From here the first set of the dependent 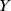 vector is calculated along with the associated error vector 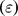. With the initial data generated the real calculations can begin.

Each loop (in which a single observation is added) first consists of calculating the posterior mean (6) and then calculating the significance of the regression parameters in 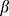 with the cycle exiting here if the number of loops reaches the target number of experiments. The mean or 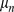 is calculated by (6). Although in this simulation we have the true values 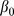, we use 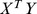 in (6) rather than using the real value or the previous mean. The significance subroutine takes the most recently calculated mean 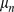 and the whole 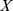 matrix. It solves for the posterior variance-covariance matrix of 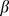 with (7) with the whole 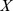 matrix and multiplies this by 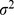. The 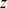-value of each 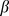 is calculated with each individual mean value divided by the square root of the diagonal of the above matrix. The p-value is then calculated using this 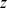-value. These 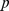-values are then sorted from largest to smallest. The resulting values are checked using a Benjamini-Hochberg method [27] to decide which components of 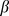 are significant. The calculation for 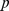-value is done using the algorithm from Press et al. [28] (on p. 221). From here, depending on the specific method in Figure 2, the new observations are calculated, and finally the new 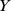 vector is calculated by 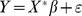 and the cycle repeats in Figure 1. The error vector 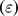 or error values are created by generating a standard normal random variable value and multiplying it by 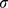 (which was set by the input file).

*MINE-like method*. The simple naïve *MINE-like method* takes the ten eigenvectors of the 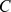 matrix in (7) associated with the ten largest 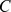-eigenvalues using a common subroutine to generate the 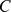 matrix and simply uses these eigenvectors to generate the next 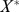.
*MINE Method*. The *MINE method* simply uses the eigenvectors associated with the 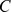 matrix as above with the ten largest eigenvalues and multiplies the corresponding eigenvectors by the square root of the 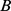 matrix to obtain the next 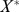.
*MINE with Random Orthonormal Basis*. In MINE with a random orthonormal basis a set of ten random orthonormal vectors is generated and then standardized by the square root of the 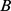 matrix 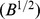. First, the ten vectors are created from using the random orthonormal set subroutine. Then each individual vector is multiplied by the square root of the 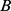 matrix to obtain the next 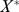.
*MINE with Random Rotation*. The MINE with a random rotation method first finds all the eigenvectors in the 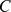 matrix as above but selects the set of all degenerate vectors instead of simply the ten largest. Then the method creates a random orthonormal array of 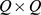 where 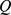 is the number of degenerate eigenvectors to multiply the degenerate matrix with. This is used to rotate the degenerate eigenvector set. The first ten are then multiplied by the square root of 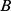 matrix and used to obtain the next 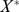.

The randomly generated orthonormal set used in both the MINE with a random orthonormal basis and MINE with random rotation is done by first generating a single vector of random Gaussian values. The vector is then normalized to a unit vector. This vector is used as the basis for generating more vectors generated in the same method and is made orthogonal using a modified Gram-Schmidt (MGS) algorithm [29].

To obtain the square root of the 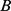 matrix, first 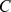 in (7) is calculated. Then the square root of 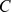 is solved by using the Singular Value Decomposition 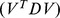 where the 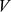 matrix here is the eigenvectors in column form and the 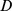 matrix is a diagonal matrix with the square root of the eigenvalues on the diagonals. This is then inverted by the method in the Jama package [25] to get the square root of 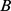 matrix.

With each of the four methods the same set of 1000 components of the true 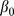 were used. This 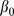 only had ten components that were truly nonzero. The order of the nonzero components was not changed in the list of 1000 components. These were the first ten values of 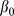 and were as follows: 11, −36, −26, 9, 33, −50, −45, 15, 3, and 17. The program was run with *1000 replicates for each of the four methods*. Each replicate had a unique pilot experiment (consisting of ten observations), but these pilot experiments were the same for each method, allowing for a stronger comparison of the four methods. Each individual run had a unique random seed so that the rest of the replicate run would be unique. The error σ in the linear model used was 0.01, and all of the prior mean values 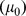 were initialized to zero. A summary of the parameters in the simulations is given in Table 1.

**Table 1:** Parameters for simulations.

n	p	d	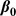	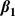	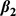	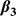	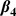	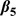	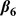	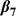	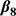	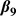	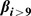	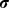
<1001	1000	10	11	–36	–26	9	33	–50	–45	15	3	17	0	0.01

## Results of Simulation

There are four variations on the MINE procedure examined here and defined in the previous section. The similarities and differences in the pathways of the four methods are summarized in Figure 2. All four methods employ the same subroutines for the majority of their implementation but differ in the way each particular method chooses the next experiment or set of observations to use, as described in the program description above.

The first method is called the naïve MINE or MINE-like method because this version does not incorporate the 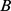 matrix used in Theorem 1. This simpler method only calculates the eigenvectors of 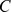 and uses the eigenvectors with the ten largest eigenvalues (according to the algorithm) to define the next experiment 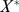. The second method, MINE, takes the MINE-like method and simply multiplies the chosen eigenvectors by 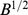. The comparison of the MINE and MINE-like method allows us to assay the importance of the standardization in the MINE procedure.

The third method, called MINE with random orthonormal basis, does not use calculated eigenvectors as suggested by Theorem 1 from, for example, matrix 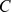. Instead, the third method creates a set of random orthonormal vectors and standardizes this basis by 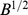. This method allows us to examine the effect of choice of the orthonomal basis in Theorem 1 on the performance of MINE. The final method tested is called MINE with random rotation. This method combines the previous methods by taking the chosen set derived in the MINE method but rotates the set by a random orthonormal basis before standardizing using a modified Gram-Schmidt Algorithm [29]. In contrast to the MINE method selecting an orthonormal basis, which is a function of the machine precision in the calculation of the eigenvectors and eigenvalues of 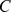, the MINE with random rotation removes this dependence on the machine precision as the “randomizer” and replaces the resulting choice of eigenvectors (axes) with a random spin of the axes defined by the eigenvectors of the 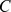 matrix.

There were a number of criteria considered for comparing the MINE methods. These criteria include (1) identifying the nonzero values of 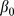 by using a Benjamini-Hochberg multiple test correction [27] at a 1% significance level, (2) identifying the correct sign and value for the nonzero components of 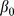, and (3) determining the number of false positives.

**Figure 3:**
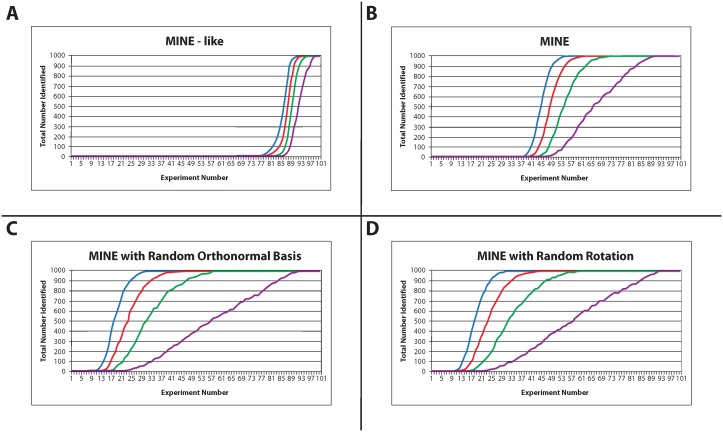
Graph of the number of significant nonzero regression coefficients averaged over 1000 replicates for: (A) the MINE-like method; (B) MINE method; (C) MINE method with random orthonormal basis; (D) MINE method with random rotation. Each graph identifies the number of replicates (y-axis) with a varying number of the nonzero 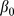 components as significant as a function of the number of experiments (x-axis). Blue corresponds to 70% correctly identified, red to 80%, green to 90% and purple to 100%.

The first criterion discerns if the methods correctly identify the nonzero values of 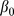 as being significant or successful discovery as given by the test described above. A method is considered better or more successful the fewer experiments are needed to discover the truly nonzero regression coefficients. In Figure 3 there are four graphs provided (A–D) to display this criterion, and all iterations performed are shown.

The MINE-like method seems to perform the poorest in this criterion (Figure 3A) in that successful discovery was very late. However, being satisfied with a lower percentage of correctly included independent variables (say 7 out of 10) allows for more replicates meeting this criterion. The method began mostly (over 50% of the replicates) identifying 7 of 10 at the 87^th^ experiment and only at the 90^th^ experiment did over 90% of the replicates identify 7 of 10 of the true components of 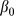. For over 90% of the replicates to identify all nonzero values of 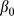 at least 97 experiments were required.

The other three methods performed significantly better than the MINE-like method. The MINE method performed almost twice as fast in this criterion as the MINE-like method (Figure 3B). For example, over 50% identified 7 of 10 at the 45^th^ experiment and over 90% at experiment 50. Only 63 experiments were required for over 90% of the replicates to identify 9 of 10 experiments. However, to get all nonzero values of 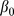 required much more time. Sixty-six experiments were needed to get over 50% and 83 experiments before over 90% of the replicates considered significant.

Both the MINE with random orthonormal basis and the MINE with rotation performed almost identically (Figures 3C and 3D). Both identify 90% of the nonzero values of 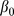 in over 900 replicates at the 47^th^ experiment. However, attempting to identify all ten nonzero components of 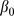 in all samples requires much more data, similar to the MINE method. It takes more than 80 experiments for both of these methods to identify all nonzero values of 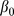 in over 800 of the replicates.

**Figure 4:**
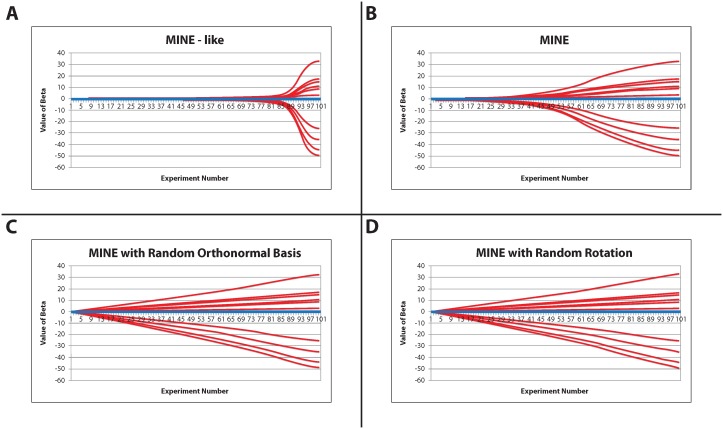
Posterior means of the first 20 regression coefficients as a function of the number of experiments for: (A) the MINE-like method; (B) MINE method; (C) MINE method with random orthonormal basis; (D) MINE method with random rotation. Each panel is averaged over all 1000 simulations with 10 zero (in blue) and 10 nonzero (in red). The first ten (red) are truly nonzero.

The second criterion involved identifying the correct sign and value for the nonzero beta values. This is evaluated by methods described earlier. Figures 4A–4D depict an average value for the first 20 values where the first ten are the nonzero coefficients and the second ten are zero and are shown as a comparison. As previously discussed, early detection is important.

As in the previous criteria the MINE-like method performed poorly (Figure 4A). After the pilot experiment the nonzero 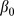 values have the correct sign identified and never change sign though the full run of experiments; however, the experimental 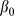 values do not reflect the components of the true vector 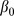 until the final experiment. Also interestingly the values increase slowly until about experiment 85 when the absolute value of each 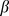 component drastically increases towards the true value.

MINE performs similarly in pattern to the MINE-like method. MINE does just as well at sign detection, with no real variable ever offering the incorrect sign (Figure 4B). The pattern of the MINE is less gradual than the MINE-like but features a slow growth then a sudden spike and approaches the asymptote of the true value. The MINE reaches the slope change between experiments 50–55 and so it takes the remaining 45–50 experiments to arrive at the plateau of the real value.

The other two methods also outperform the MINE-like method. Again, in this criterion the MINE with random orthonormal basis and the MINE with random rotation perform almost identically (Figures 4C and 4D). Sign identification seems to be the easiest criterion as these two also perform flawlessly here. Unlike the previous two, these methods seem to have a very linear pattern in the values of the 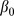 nonzero components.

**Figure 5:**
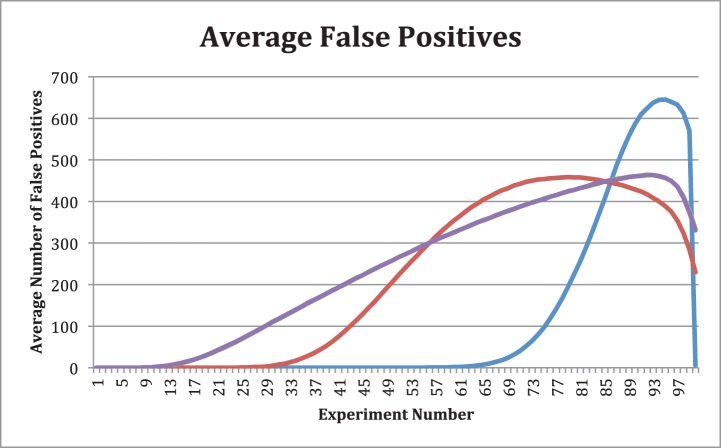
The number of false positives as a function of the number of experiments. These numbers are averaged over all 1000 simulations for each method. Blue corresponds to MINE-like, red to MINE, green to MINE with random orthonormal basis, and purple to MINE with random rotation. The final two overlap almost exactly which is why the green line is not visible.

The next criterion involves comparing the false positives of each method (Figure 5). A false positive happens if any of the 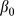 that are actually zero are considered significant. For comparison, the average number of incorrectly identified values, averaged over all simulations for each method, is shown.

Again, we see similar patterns where the MINE-like method performs poorest. Initially, it looks like it is performing adequately, since up until experiment 60 there are zero false positives. Since we have to wait until experiment 85 for any reasonable amount of success, we find that the false positives are beyond 400 and at some of the highest peaks compared to the other three methods. The only way this method could possibly be considered better is that it drops off faster at the final observed experiment but since this point is a worst case, this point should not be reached.

The MINE method has a similar pattern to the MINE-like, again performing in a similar scaled manor. At the point where information could be accepted for the nonzero values, around experiment 55, the false positive rate is around 300 which would allow for about 70% of the variables to be eliminated. If more experiments are performed, the number identified peaks just under 460 during experiments 73–84 afterwards it gradually begins dropping.

The MINE with random orthonormal basis and MINE with random rotation again display almost identical results. However, in contrast to the MINE-like and MINE methods, these two have a more linear growth of false positives, especially after the first 15 and before the last 15 experiments. Due to the data being displayed on a single graph, the similarities are more observable with the two lines eclipsing each other. During the most optimal selection periods between experiments 20–45, the false positives do not go over 225. This allows for a greater than 75% reduction in variables. These two do however peak ever so slightly higher with the average reaching just under 465 but a much later experiment and are only lower during a 20–30 experiment window. All simulation results in Figures 3–5 are summarized in an excel file generated with the program MINE under the keyword linearminesimulations at sourceforge.net.

Having determined that the MINE with random rotation and MINE with random orthonormal basis perform similarly and outperform the other two procedures, we conclude by examining the properties of the MINE with random rotation, namely its power and false positive rate, in a situation with increased noise in Table 2. Not unexpectedly with more noise it takes a larger number of experiments before the power to detect 7 out of 10 true regression coefficients are significant is large as the noise (measured by 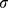) is increased. The false positive rate is controlled when one stays below the number of experiments necessary to obtain 7 out of 10 true regression coefficients most of the time. As an example, for a 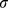 of 0.05 and a power of 98.3% in Table 2, the false positive rate is only 0.94%.

**Table 2:** The effects of varying the noise level 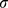 on the power to detect 7 out 10 of the truly nonzero regression coefficients and the false positive rate for the MINE with random rotation.

		number of experiments					
σ	%	30	40	50	60	70	80
.01	100×power to detect 7/10 regressioncoefficients	99.1%	99.9%	100%	100%	100%	100%
.01	100×false positive rate	10.9%	18.6%	25.9%	32.5%	38.2%	42.8%
.05	100×power to detect 7/10 regressioncoefficients	0.1%	0.2%	0.2%	0.2%	23%	98.3%
.05	100×false positive rate	0.5%	1.05%	1.09%	0.88%	0.60%	0.94%
.10	100×power to detect 7/10regression coefficients	0%	0%	0%	0%	0%	0.2%
.10	100×false positive rate	0.9%	0%	0%	0.02%	0.01%	6.73%

## Discussion

In 2008 a key problem in systems biology was solved as identified by Kitano [30] with a new methodology called MINE [4]. The MINE methodology is used to integrate several cycles of modeling and experiments to yield discoveries about the underlying process being studied. The result of the application of the MINE methodology was new insights into the relation of the clock to ribosome biogenesis [1], [4]. This new approach to model-guided discovery has sparked a flurry of developments in MINE methodology [19], [31], [32], [33]. It is natural to ask how this new experimental design methodology of MINE is related to classical experimental design criteria and whether or not we can validate MINE mathematically as a discovery tool when there are many parameters and sparse, noisy data 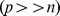. A natural place to validate this new MINE tool is in the framework of the oldest and mostly widely used statistical model, the linear model.

One of the consequences of the work here is to establish another view of one MINE procedure. When the same constraints are imposed on MINE and the D-optimality criterion, then the MINE procedure discussed here is D-optimal under the linear model. The effect of minimizing the determinant of the correlation matrix of the predictions is equivalent to minimizing the determinant of the variance-covariance matrix of the parameter estimates as described in detail in the Equivalence Theorem 2 when the same constraints are imposed on both problems. We suspected this would be the case from the application of the MINE procedure in systems biology, where the application of the MINE procedure appeared to decrease the estimated error variance 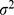 over time [4]. In the language of the microscopy analogy, maximizing the volume observed under the microscope by choice of experiment is equivalent to reducing the ellipsoid of variation in the optical field of the parameter space. It is this key relation that Marvel and Williams exploit to address Kitano’s problem [19].

Having shown the MINE procedure in practice is useful for discovery [4], it is natural to ask how MINE performs in a simpler setting of the linear model. We explored its performance under four variations. In this simpler setting, where we can actually calculate the ensemble directly without resorting to using Markov Chain Monte Carlo as used in nonlinear systems [34], we can solve the associated optimization problem of MINE in Theorem 1 in a way that may suggest new approaches to MINE in nonlinear models. The result of Theorem 1 was the realization that the maximization of the MINE criterion here is defined up to an orthonormal basis of the data space. There are a variety of different bases that could be selected. Theorem 1 also calls for a standardization of the basis. This standardization does prove important as we see upwards of a 50% improvement in some criteria between the MINE-like and the MINE methods.

First, it was important to see the two more similar methods (MINE with random orthonormal basis and MINE with random rotation) performed similarly. Second, these two proved better in all of the criteria in almost all experiments. These allowed for the earliest detection, during which they provided the closest to true values on all variables, and provided the fewest false positives for a larger sample reduction. The only area at which these two methods were out performed was in the number of experiments needed for most of the simulations to identify the real values given that initial detection had begun. The MINE method once 10% of the simulations began detecting these values was able to reach 90% more quickly. Though this region was smaller for the MINE method, the other two were not only able to reach or arrive at the 10% quicker but generally complete (get over 90%) quicker.

A third consequence of this work is to open up a new convex programming problem that is closely tied to the max det problem so thoroughly analyzed by Boyd and co-workers [22]. The argument here in the max det problem is quadratic in the design parameters with linear inequality constraints potentially as opposed to an affine argument. An open question is whether or not this new problem is a convex programming problem. If so, then much of the machinery developed by Boyd and coworkers could be developed for the problem here. We have illustrated the use of the convex programming procedure in our discussions in this work.

In conclusion, we feel that the MINE discovery tool has opened up many exciting design problems that will transform the way scientists now integrate theory and experiment in a number of areas beyond systems biology [3], [35], [36].
